# Does DeepSeek curb the surge of energy consumption in data centers?

**DOI:** 10.1016/j.xinn.2025.100944

**Published:** 2025-05-09

**Authors:** Yongzhen Wang, Yibo Han, Kai Han, Jun Shen

**Affiliations:** 1 School of Mechanical Engineering, Beijing Institute of Technology, Beijing 100081, China; 2 Beijing Laboratory for System Engineering of Carbon Neutrality, Beijing Institute of Technology, Beijing 100081, China; 3 Innovation Center in Chongqing, Beijing Institute of Technology, Chongqing 401120, China; 4 Yangtze Delta Region Academy, Beijing Institute of Technology, Jiaxing 314019, China

## Main text

The extensive utilization of large language models (LLMs) within the artificial intelligence (AI) domain, such as ChatGPT and Sora, has driven a massive demand for computing power, thereby necessitating a stable and continuous supply of electricity. Consequently, the energy consumption of data centers (DCs) has surged significantly over the past few years. Recently, the emergence of highly efficient models, exemplified by DeepSeek, has had a considerable impact on the industry, driven by breakthroughs in algorithms, architectures, and various other technologies. The increasing recognition and widespread application of these AI models are predicted to have a profound impact on both the demand for computing power and the energy consumption of DCs, which has become a central concern for energy professionals and policymakers.

**Figure 1:**
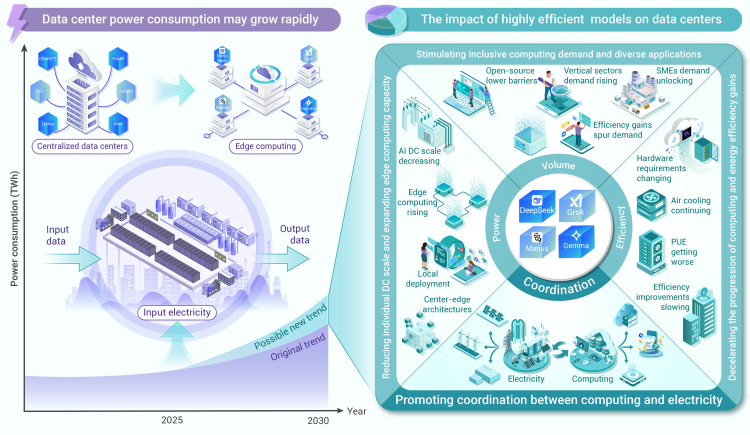
The growth trend and driving forces of energy consumption in DCs under the technological advancement of DeepSeek

Accordingly, as shown in Figure 1, this paper attempts to elucidate the potential impacts of efficient models on the energy consumption of DCs from the perspectives of inclusive computing power, electricity usage scale, energy efficiency, and the coordination between computing and electricity.

## Stimulating inclusive computing demand and diverse applications

In the realm of AI demand, highly efficient models exhibit a counterintuitive phenomenon outlined by the Jevons Paradox: while technological advancements enhance individual energy efficiency and cost-effectiveness, they can paradoxically lead to an overall increase in energy consumption due to extensive utilization. Firstly, the rapid surge in DeepSeek users has led to frequent occurrences of “servers busy, please try again later” messages, underscoring the robust market demand and the necessity for continuous infrastructure expansion to accommodate the growing user base. Secondly, AI has matured in various common applications, including text generation, image and video creation, as well as conversational and search functionalities. But now an increasing number of industries and scenarios, such as healthcare, transportation, and manufacturing, are experiencing a significant surge in AI-related applications.^1^ This trend contributes to an exponential rise in both training and inference demands, inevitably driving up resource consumption. Thirdly, breakthroughs in highly efficient models have substantially reduced the marginal cost of training. Previously, the high costs associated with computing power constrained the ability of startups and small enterprises to innovate in AI. However, the removal of this cost barrier has unleashed previously suppressed market demand, leading to a proliferation of diverse applications across various sectors.

## Reducing individual DC scale and expanding edge-computing capacity

DeepSeek achieves high performance through algorithmic and architectural innovations, enabling comparable performance with fewer high-end servers and lower energy consumption. This effectively reduces reliance on large centralized DCs and alleviates overall electricity demand. Traditional centralized DCs typically require power supplies of nearly hundreds of megawatts. In contrast, highly efficient models decrease the computing power demands for both training and inference. Consequently, even small-scale edge DCs can operate with power requirements of only tens of kilowatts. Therefore, this development will prompt the industry to reevaluate the scale and design of DCs. While the number of large-scale DCs may not decline immediately, their previous rapid growth trend is expected to significantly slow down. Meanwhile, the size of small-scale edge DCs is anticipated to increase rapidly, like small intelligent-computing devices of various types that integrate training and inference.

Furthermore, the deployment of streamlined AI models on local devices and at edge DCs has become an emerging trend, a process that DeepSeek may accelerate. For instance, DeepSeek-R1 claim to deliver powerful inference capabilities on devices ranging from laptops to embedded systems. This shift not only necessitates that DCs provide cloud computing support but also facilitates collaborative training and updates of pre-trained model at the edge. It is reasonable to envision that a hybrid computing architecture integrating centralized and edge computing will become increasingly prevalent. Centralized facilities will primarily handle the training of LLMs and complex inference tasks, while edge nodes will focus on real-time, local inference to meet the ever-growing and diverse demands of AI. This shift may imply that the total energy consumption of a substantial number of small and medium-sized, as well as edge, DCs will exhibit a relatively rapid growth trend.

## Decelerating the progression of computing and energy-efficiency gains

Compared to traditional CPU-based DCs, AI DCs typically integrate high-performance GPUs and other specialized accelerators, which exhibit significantly higher server power and thermal-flux density.^2^ However, highly efficient models may reduce the number of GPU parameters required for training and enable DC owners to transition toward using mid- to low-end GPUs. In fact, the power consumption per unit of performance of mid- to low-end servers is generally lower than that of high-end servers. Consequently, this not only lowers the investment costs of servers but also simplifies the thermal management of servers, enabling traditional air-cooling systems to remain viable and delaying the widespread adoption of more costly liquid-cooling technologies. Therefore, in the short term, the power consumption per unit of computing power in DCs may not necessarily decrease; it may even increase.

In addition to changes in server power consumption, the energy consumption of cooling systems for heat dissipation is also a significant factor in DCs. While hybrid center-edge computing architectures facilitate real-time inference, they also tend to stimulate increased computing demand in smaller DCs. These smaller facilities typically exhibit higher power usage effectiveness (PUE; the ratio of the total energy consumed by a DC to the energy consumed by its IT equipment). At the same time, smaller DCs may not employ more advanced IT equipment, resulting in suboptimal performance metrics for indicators such as IT equipment utilization and IT equipment energy efficiency. Overall, although efficient AI models reduce energy consumption on a per-task basis, their widespread adoption could ultimately constrain the overall efficiency gains of DCs by stimulating broader computing demand, particularly in smaller DCs.

## Promoting coordination between computing and electricity

With advancements in semiconductor technology and market expansion, the cost per unit of computing power continues to decline, driving the proliferation of AI applications. Although highly efficient models reduce the deployment costs of IT equipment, the surge in demand for computing power will lead to a sharp increase in DCs’ energy consumption. Electricity expenses account for as much as about 60%–70% of the operating expenditures of DCs, directly impacting the profitability of DC owners. As DeepSeek accelerates the democratization of IT investment and makes computing power services cheaper, the proportion of power operating costs relative to the total investment or the revenue from computing power sales in DCs is expected to rise further (here, we might introduce a new metric, such as the ratio of the unit price of computing power services to the price of electricity supply). Consequently, fluctuations in energy prices are predicted to have a direct and profound impact on the cost structure of DCs. Ultimately, both the supply and costs of electricity will significantly influence the spatiotemporal distribution as well as the construction and operational characteristics of various computing tasks within DCs.

These changes will facilitate the adoption of various electricity pricing mechanisms to promote a computing-electricity coordination pattern, providing new momentum and demonstration of effects for the green and low-carbon transformation of DCs. Unlike the energy-consumption characteristics of other industries, such as steel and cement production, data-computing tasks can be relatively easily and dynamically adjusted according to electricity prices.^3^ The computing-electricity coordination pattern implies that DCs can achieve dynamic interaction with the new power system, flexibly adjusting electricity usage strategies based on grid load and price signals. Through the adoption of an innovative integrated energy system model for DCs, optimized strategies for the low-cost and environmentally friendly operation of DCs can be identified, thereby enhancing the utilization of wind and solar renewable energy in the region.^4^

Future developments in the computing power market may include flexible financial models. These models allow enterprises to reserve future computing power or purchase idle computing power during off-peak periods for demand, which further improves resource utilization. Implementing such models undoubtedly requires mature computing power trading platforms and standardized measurement systems. The government and the industry may encourage the establishment of a computing-power trading market, thereby achieving market-based pricing and diversifying revenue streams for intelligent-computing centers. Here, we can use the concept of “one kilowatt-hour of electricity” to measure computing power.^5^ This initiative will standardize the computing power settlement standards, which will not only lay a commercial foundation for resource interconnection but also enhance the flexibility of resource allocation, reduce the construction and operation costs of computing power, and thus promote the widespread application of computing-electricity coordination patterns.

## Conclusion

Overall, we believe that the global AI power demand, driven by models such as DeepSeek and Manus, will sustain a relatively rapid growth trend. However, numerous uncertainties remain under active debate. In this wave of the democratization of computing power, it is crucial to explore the integration of efficient AI technologies with existing energy infrastructures and to promote new patterns, such as computing-electricity coordination and computing-thermal coordination. By leveraging intelligent scheduling, price incentives, and coordinated optimization of power and computing markets, efforts can be directed -power achieving a dynamic balance between enhanced computing performance and reduced overall energy consumption. This approach will provide robust technical support and strategic guidance for the green, low-carbon transformation of DCs and the broader energy system.

Future research should concentrate on improving life-cycle assessments to evaluate the life-cycle energy and carbon impacts of highly efficient AI models, refining long-term forecasts of AI energy demand, and optimizing workload scheduling for sustainable grid integration. Additionally, it is essential to develop a comprehensive evaluation framework that encompasses green-power adoption, computing efficiency, and auxiliary energy use. Finally, enhancing the coordination between computing and electricity markets will enable computing power to become a dynamic and tradable resource.

## Funding and acknowledgments

This work is supported by the 10.13039/501100001809National Natural Science Foundation of China (project no. 52006114).

## Declaration of interests

The authors declare no competing interests.
